# Tracking Cats: Problems with Placing Feline Carnivores on δ^18^O, δD Isoscapes

**DOI:** 10.1371/journal.pone.0024601

**Published:** 2011-09-09

**Authors:** Stephanie J. Pietsch, Keith A. Hobson, Leonard I. Wassenaar, Thomas Tütken

**Affiliations:** 1 Zoologisches Forschungsmuseum Alexander Koenig, Universität Bonn, Bonn, Germany; 2 Environment Canada, Saskatoon, Saskatchewan, Canada; 3 Steinmann-Institut für Geologie, Mineralogie und Paläontologie, Universität Bonn, Bonn, Germany; Roehampton University, United Kingdom

## Abstract

**Background:**

Several felids are endangered and threatened by the illegal wildlife trade. Establishing geographic origin of tissues of endangered species is thus crucial for wildlife crime investigations and effective conservation strategies. As shown in other species, stable isotope analysis of hydrogen and oxygen in hair (δD_h_, δ^18^O_h_) can be used as a tool for provenance determination. However, reliably predicting the spatial distribution of δD_h_ and δ^18^O_h_ requires confirmation from animal tissues of known origin and a detailed understanding of the isotopic routing of dietary nutrients into felid hair.

**Methodology/Findings:**

We used coupled δD_h_ and δ^18^O_h_ measurements from the North American bobcat (*Lynx rufus*) and puma (*Puma concolor*) with precipitation-based assignment isoscapes to test the feasibility of isotopic geo-location of felidae. Hairs of felid and rabbit museum specimens from 75 sites across the United States and Canada were analyzed. Bobcat and puma lacked a significant correlation between H/O isotopes in hair and local waters, and also exhibited an isotopic decoupling of δ^18^O_h_ and δD_h_. Conversely, strong δD and δ^18^O coupling was found for key prey, eastern cottontail rabbit (*Sylvilagus floridanus*; hair) and white-tailed deer (*Odocoileus virginianus*; collagen, bone phosphate).

**Conclusions/Significance:**

Puma and bobcat hairs do not adhere to expected pattern of H and O isotopic variation predicted by precipitation isoscapes for North America. Thus, using bulk hair, felids cannot be placed on δ^18^O and δD isoscapes for use in forensic investigations. The effective application of isotopes to trace the provenance of feline carnivores is likely compromised by major controls of their diet, physiology and metabolism on hair δ^18^O and δD related to body water budgets. Controlled feeding experiments, combined with single amino acid isotope analysis of diets and hair, are needed to reveal mechanisms and physiological traits explaining why felid hair does not follow isotopic patterns demonstrated in many other taxa.

## Introduction

Many carnivore species are currently threatened and are the focus of intense conservation concern [1]. Feline carnivores are often subject to illegal wildlife trade, thus the ability to estimate the geographic provenance of illegal tissue samples would constitute important information in wildlife crime investigations [2]. Probabilistic provenance determination based on O and H isotopes has strong potential to be applied to animal tissues as an investigative tool in wildlife forensic science [3]–[6]. Validation of isotopic methods has relevance and practical application in various fields like wildlife forensics and conservation biology.

Measurements of the stable isotopes of hydrogen (δD) and oxygen (δ18O) of animal keratinous tissues have been used to track the geographic origin and migratory patterns in a wide variety of animals (e.g. [3], [4], [7]–[9]). To date, this approach is based on strong empirical correlations between δD values in animal tissues (δD_t_) with the isotopic composition of the amount-weighted mean annual or mean-growing season precipitation (δD_p_). The latter correlates inversely with latitude and elevation across the continents, especially in North America [10]–[12]. Few studies have coupled δD and δ^18^O measurements of the organic or inorganic fractions of animal tissues despite the strong covariance between these isotopes in environmental waters (hairs and nails: human [8], [13]–[16]; CO_2_, body water, hair and enamel: woodrat [17]; chitin: brine shrimp [18]; chitin: chironomids [19]; plasma, blood and feathers: birds [20], [21]; fat, blood, muscle, hair and collagen: pig [22]; carbonate and phosphate tooth enamel, bone collagen, subcutaneous fat and hair: laboratory rat [23]). Strong correlations between δD_p_ and δD_t_ have been found for many species [4]. The hydrogen and oxygen isotopic composition of animal tissues (hair, feathers, teeth) is related to the isotopic composition of body water (e.g. [24]–[27]) and ultimately to that of ingested water. Influences on isotopic composition of body water (δD_bw_, δ^18^O_bw_) of animals include abiotic (climate, drinking water) and biotic (diet and physiology) factors [28]–[35]. The incorporation of H and O isotopes from the hydrosphere via diet and drinking water into animal tissues is a complex process and our understanding of how these mechanisms affect the nature and variability of the empirically observed relationships is still poor (e.g. [13]). However, to reliably track the geographic origin of an animal requires a detailed understanding of the metabolic routing of dietary nutrients and mechanisms of H and O isotopic incorporation into animal tissues [36].

Hydrogen and oxygen in animal tissues can be derived from two potential sources: dietary nutrients and body water, whereas oxygen is also derived from inhaled air. The body-water pool, in turn, is derived from ingested drinking-, food-, and metabolic-water produced during the catabolism of food macromolecules [28], [30], [32], [35], [37]–[39]. The relative contributions of all these sources to protein synthesis (i.e. keratin and collagen) are likely to vary among animals [40]–[42]. Controlled experiments are key to understand and model the incorporation of H and O isotopes into proteinaceous tissues like keratins (hair and feathers), collagen, and chitin, and have so far been developed for only a small number of species like woodrat (*Neotoma cinerea* and *Neotoma stephensi*; [17]), rat (*Rattus norvegicus*; [23]), Japanese quail (*Coturnix japonica*; [24]), house sparrow (*Passer domesticus*; [21]), humans (*Homo sapiens*; [8], [13]–[16], [27]), pig (*Sus scrofa domesticus*; [22]), brine shrimp (*Artemia franciscana*; [18]) and chironomids (*Chironomus dilutus*; [19]). These studies revealed that keratin δD and δ^18^O reflect both biological (diet, physiology) and environmental signals (water, geographic movement, climate; [13]). Deviations from a strong coupling between δD_t_ and δD_p_, and δ^18^O_t_ and δ^18^O_p_ have been shown (e.g. [13], [43]) and may be linked to: 1) climatic factors like relative humidity [37], [44]; 2) isotopic disequilibrium of food and water contributions to δD_t_ [27]; 3) possible trophic-level effects on δD_t_ [45]; 4) impacts of metabolic rate and drinking water flux on δD_bw_ and δ^18^O_bw_ [26], [28], [30], [32] (δ^18^O of phosphate in urinary stone [46], bone [25] and tooth [47]); and 5) dietary and physiological controls on δ^18^O_h_ and δD_h_ of hair [13].

Previous studies that successfully applied combined δD_t_ and δ^18^O_t_ analysis to track the geographic origin and migration of animals focused on herbivores and omnivores (e.g. [3], [9], [17], [21], [22], [24]). The fact that this method performs particularly well in omnivorous modern humans [8], [13]–[16], [48] is not surprising, because humans are well-hydrated and typically consume a constant local water source (e.g. tap water: [49]–[51]) and consistent homogenous diet across regions (e.g. fast food: [52]). But even for humans, hydrogen isotopic incorporation during keratin synthesis likely varies between different keratinous tissues like nail and hair [53]. Free-ranging carnivores, however, differ significantly in their nutritional, physiological and metabolic characteristics from herbivores and omnivores [54], [55]. The house cat, *Felis catus*, is the most thoroughly studied mammalian carnivore [54]. Felids are strict carnivores and thus obtain much of their body water from the consumption of prey [54]. Owing to the lack of empirical H/O isotope studies on strict carnivores (other than raptors) it is unclear whether carnivore hairs track the spatially predictable meteoric water signal (despite their integrative high trophic position). However, Kohn [30] hypothesized, that “carnivore bone phosphate should track the meteoric water signal more closely than do herbivores”. For this reason, the concept of geographic source determination based on H/O isotopes using carnivore hairs as an investigative tool in wildlife forensic science needs to be tested.

Here, we provided the first large-scale δD and δ^18^O analysis of hair samples from wild individuals of two North American feline carnivores, bobcat (*Lynx rufus*) and puma (*Puma concolor*). Both species were ideally suited to test the strength of the isotope approach in assigning geographic origins of felidae. The availability of skins from museum collections, high-resolution precipitation δ^18^O and δD isoscapes for North America and ecological differences between these study animals (e.g. body size, home-range size, habitat use, distribution and prey preferences) allowed us to assess the application and efficacy of H/O isotope fingerprinting for forensic spatial assignment in feline carnivores.

Our study was designed to determine whether puma and bobcat hairs varied predictably in their isotopic composition among isotopically distinct geographic locations and reflected the spatial pattern of isotopic variation in precipitation. Furthermore, we examined if species- or sex-specific effects existed, and whether these could be explained by differences in diet, body size and foraging ecology. Our results demonstrated that the application of water isotopes for provenance determination of feline carnivores was compromised by major controls of their diet, physiology and metabolism on δ^18^O_h_ and δD_h_. The controlling factors and possibilities to quantify these will be discussed.

## Materials and Methods

### Ethics statement

All CITES permits (MA 125284-0) for the export and use of museum materials from puma and bobcat were issued by the U.S. Fish and Wildlife Service.

### Study species and sampling

**Figure 1:**
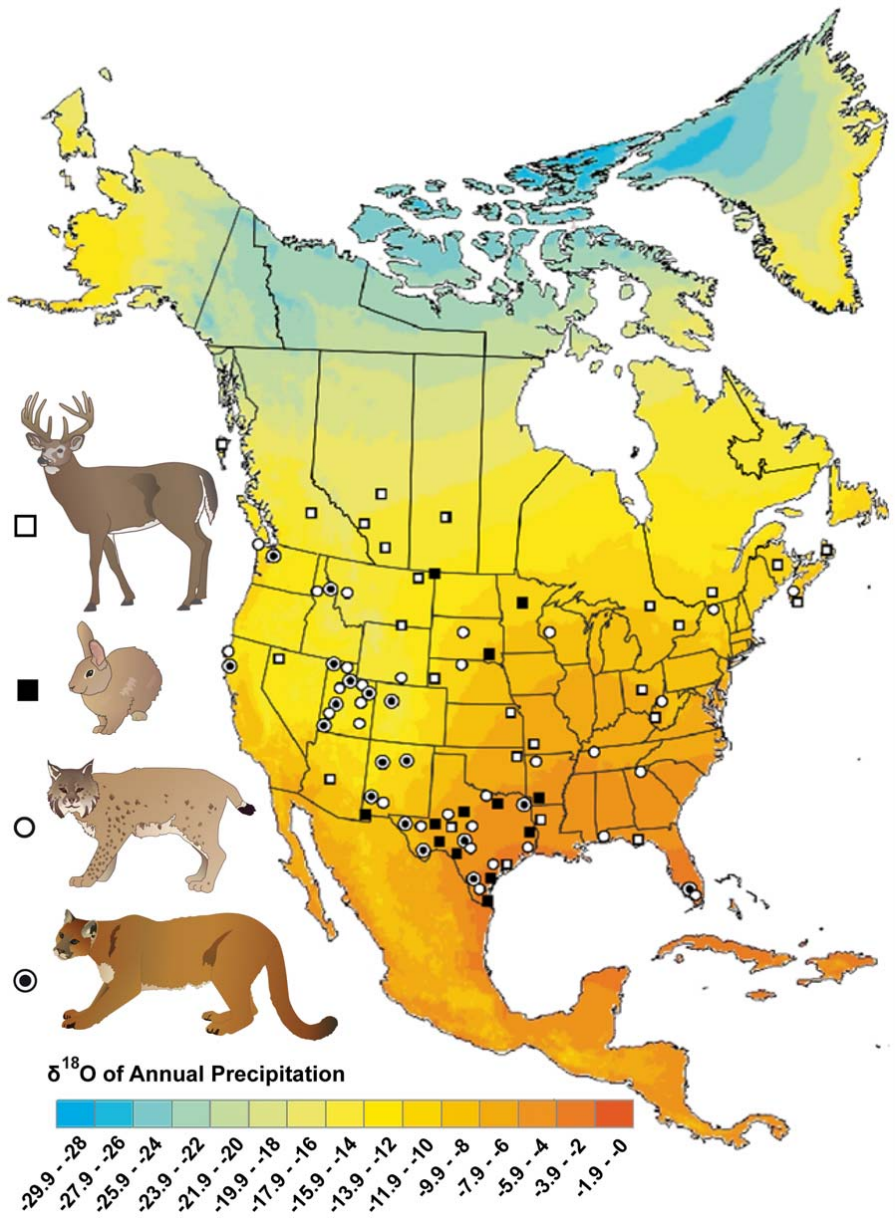
Map of sampling sites. Sample locations for both felines bobcat (n = 45) and puma (n = 30) as well as their preferred prey species eastern cottontail rabbit (n = 13) and white-tailed deer* (n = 31), respectively, plotted on the δ^18^O precipitation map of North America** (*data from [56]; **from http://www.waterisotopes.org).

Eighty-eight hair samples from two North American felid species bobcat (*Lynx rufus*, n = 45) and puma (*Puma concolor*, n = 30), as well as the eastern cottontail rabbit (*Sylvilagus floridanus*, n = 13), the latter representing the preferred prey species of the bobcat, were obtained from the Smithsonian National Museum of Natural History in Washington D.C. and the Utah Museum of Natural History, Utah. Published isotope data of bone-phosphate (δ^18^O_bp_) and bone collagen (δ^18^O_bc_) from white tailed deer (*Odocoileus virginianus*), constituting the major prey of the puma, were included for comparative analysis [56]. For each specimen, geographic location, sex and elevation was recorded (Table S1). All specimens studied originated from 75 different sites across the United States and Canada (Figure 1). Sample locations ranged in latitude from 25.8 to 48.2°N and longitude from 124.4 to 65.8°W, covering strong altitudinal (2 to 3400 m) and isotopic gradients (δ^18^O_riv_  =  −17.5‰ to −0.1‰; δD_riv_  =  −132.7‰ to 0.6‰).

### Stable isotope analysis

Sample preparation and H/O isotope analysis were conducted at Environment Canada. All keratin samples were physically cleaned of adhering debris and washed twice in a 2∶1 mixture of chloroform and methanol to remove lipids from the keratin surface. After cleaning, all samples were air-dried for 24 h. Hair samples were then cut into 0.5 cm increments (H: 350±20 µg; O: 700±50 µg) and weighed into pre-combusted silver foil capsules for H and O isotope ratio analysis. For δD, in order to account for exchangeable hydrogen in hair proteins, we used comparative equilibration with in-house keratin working standards, BWB (−108‰), CFS (−147.7‰), CHS (−187‰), for which the δD value of non-exchangeable H had been previously established [57]. For δ^18^O, we used the IAEA benzoic acid standards IAEA 601 and 602, with assigned δ^18^O values of +23.1‰ and +71.4‰, respectively. For H/O isotopic analyses, samples and reference materials were separately pyrolyzed on a Hekatech HTO elemental analyser at 1350°C to H_2_ and CO for isotopic analysis on an Isoprime™ dual-inlet isotope-ratio mass spectrometer. The reference standards were used to normalize unknown samples to the Vienna Standard Mean Ocean Water-Standard Light Antarctic Precipitation (VSMOW-SLAP) standard scale [57].

### Estimates of drinking water isotope compositions (δD, δ^18^O) for bobcat and puma

The H and O isotopic composition of water ingested by both felid species indirectly from their prey were inferred from modeled isoscape values [58] as well as measured river water values across North America [59], [60]. It was assumed that the place of death of each puma and bobcat reflected their lifetime habitat. For each locality the average δD and δ^18^O values for precipitation were determined using the Online Isotopes in Precipitation Calculator (OIPC) version 2.2 (http://www.waterisotopes.org). The OIPC provided a model estimation of long-term annually or monthly averaged precipitation isotope ratios at specified locations through spatial modelling of a large database of precipitation isotopic data covering the time period 1960–2004 [10], [58]. The δD and δ^18^O data of the OIPC model were compared to those measured for local river waters [59], [60]. In general, there was a good correlation between δD_riv_ and δ^18^O_riv_ and δD_p_ and δ^18^O_p_ for relatively small- to medium-sized drainage catchments (<130,000 km^2^) [9]. As puma and bobcats have smaller home-range sizes (female bobcat: 21.7 km^2^, [61], [62]; female puma 175.8 km^2^, [61]) local river water should reflect the average δD and δ^18^O values of ingested prey-derived drinking water. Therefore we compared the hair δD_h_ and δ^18^O_h_ data with the river water data.

Bobcat and puma hair isotope values were plotted against amount-weighted long-term annual, spring (three months mean of March, April, May) and summer (three months mean of June, July and August) precipitation δD_p_ and δ^18^O_p_ values, because the formation and isotopic incorporation of cat hair is limited to a rather short time period. For instance hair growth in domestic cats is not continuous [63], but rather includes an anagen phase of active growth and a telogen phase of rest [64]. The hair-growth phase takes 6–8 weeks and 70% percent of the hair follicles are in the anagen phase during the summer [65]. Isotopic signals from drinking water and prey consumed during the anagen phase of growth are most likely integrated into the growing hairs. For this reason we related the isotope values of hair δD_h_ and δ^18^O_h_ not only to annual average δD_p_ and δ^18^O_p_ values but also to seasonal spring and summer precipitation to test if a better relation with water isotope values of the likely main hair growing season was obtained (Table S2).

### Statistical analysis

**Figure 2:**
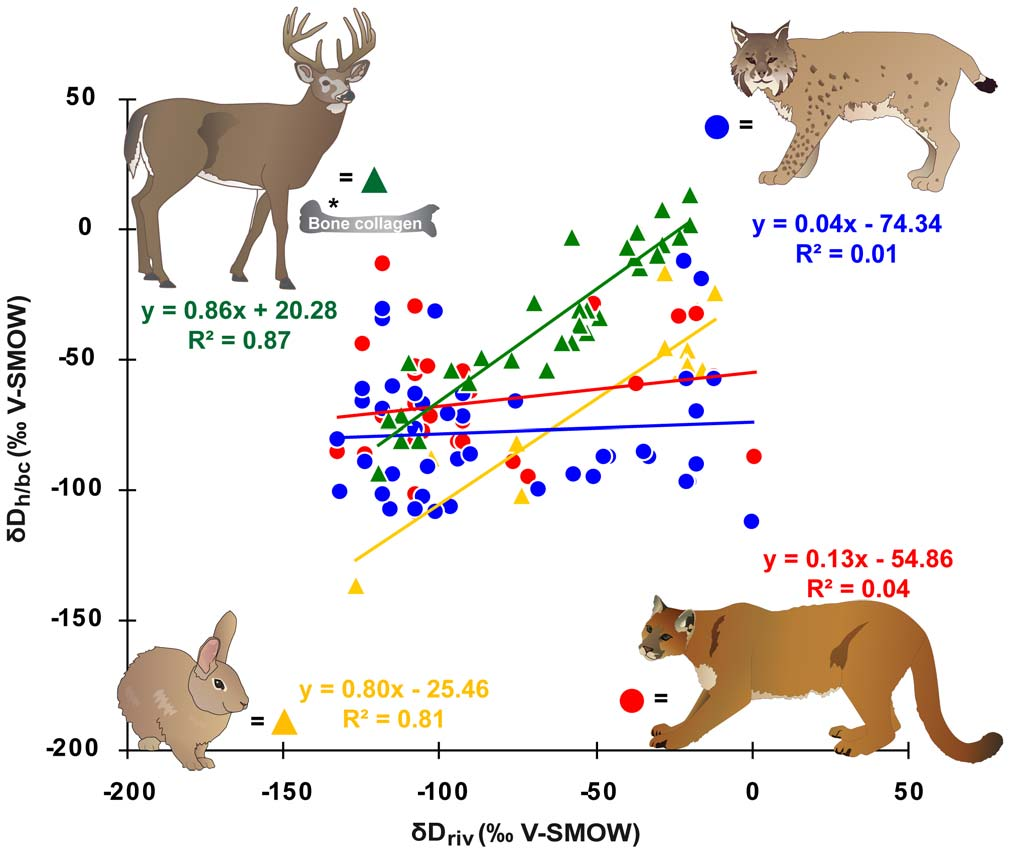
Hydrogen isotope values of keratin relative to river water. Plot of δD of hair (δD_h_) from bobcat, puma and eastern cottontail rabbit as well as bone collagen (δD_bc_) from white-tailed deer* vs. mean annual δD of river water (δD_riv_) (*data from [56]).

**Figure 3:**
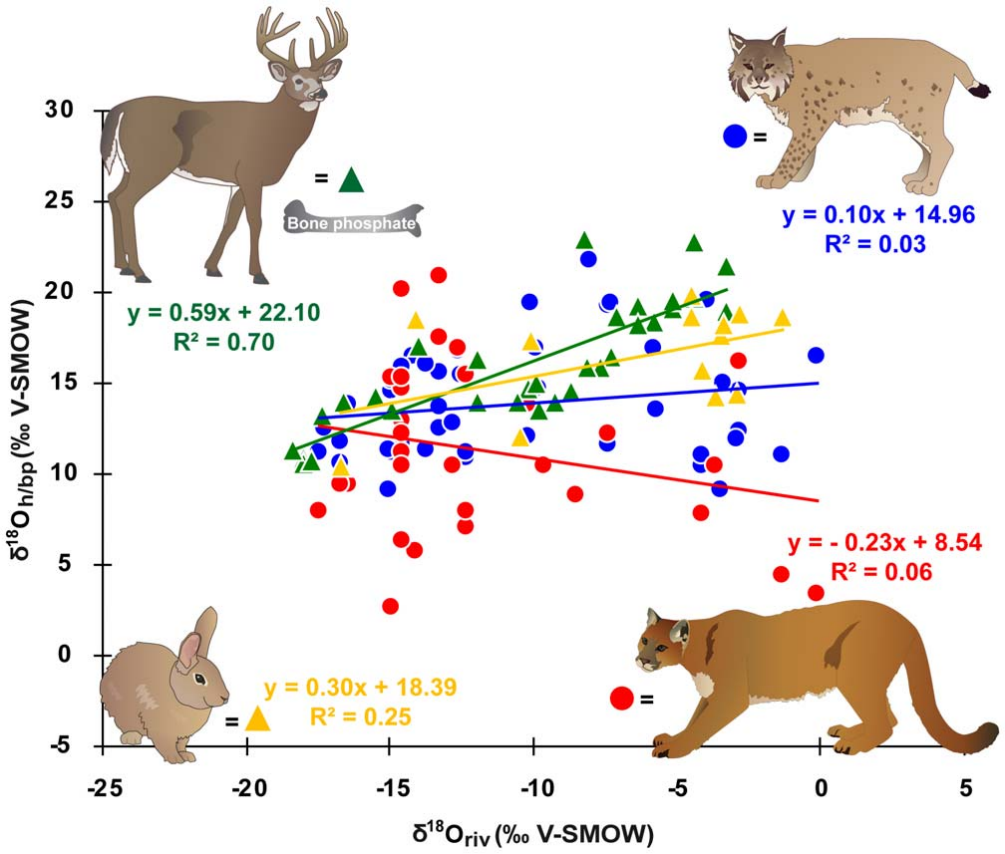
Oxygen isotope values of keratin relative to river water. Plot of δ^18^O of hair (δ^18^O_h_) from bobcat, puma and eastern cottontail rabbit and bone phosphate (δ^18^O_bp_) from white-tailed deer* vs. mean annual δ^18^O of river water (δ^18^O_riv_) (*data from [56]).

**Figure 4:**
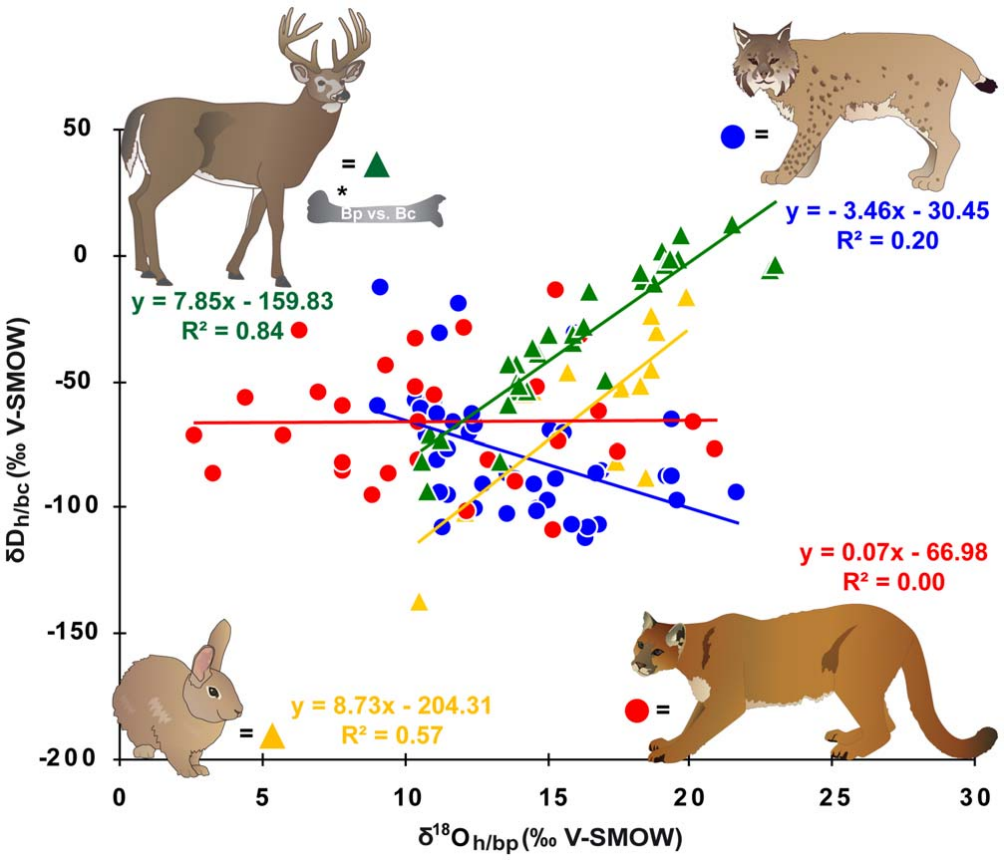
Hydrogen and oxygen isotope ratios of keratin. Hydrogen and oxygen isotope compositions are shown for hair samples (δD_h_, δ^18^O_h_) from puma, bobcat and eastern cottontail rabbit as well as collagen (δD_bc_) and bone phosphate (δ^18^O_bp_) data from white-tailed deer* (*data from [56]).

First, we analysed the H and O isotopic variation of puma and bobcat hairs among locations and their correlation with the large-scale patterns of isotopic variation in precipitation. We tested whether the correlations significantly changed when using the annual and summer modeled precipitation or local river water data (Table S2). We compared hair H and O isotope data of predators and respective prey species and tried to establish a calibration equation between river water and hair for a feline carnivore. Relationships between mean annual δ^18^O_riv_, δD_riv_ and δ^18^O_h_, δD_h_ of puma, bobcat and rabbit hairs were investigated using linear regressions (Figure 2 and 3). We also examined the relationship between v^18^O_h_ and δD_h_ (Figure 4). The effects of species, age, sex, seasonal precipitation and relative humidity on hair isotope values were examined using a General Linear Model (GLM) (Table S2). Statistical tests were conducted using XLSTAT (V 7.5.2).

## Results

All hair δD_h_ and δ^18^O_h_ values were plotted against mean annual δD_riv_ and δ^18^O_riv_ values because using either amount-weighted mean annual, summer (June, July and August) or spring (March, April and May) OIPC modeled precipitation values did not significantly change the results (Table S2). The δ^18^O_h_ - δ^18^O_p_ correlation of bobcats was slightly improved by including relative humidity in the regression (R^2^ = 0.21, p = 0.01, n = 44). Relative humidity did show a significant modest effect on δ^18^O_h_ of bobcats (R^2^ = 0.21, p = 0.002, n = 44) but no effect on δ^18^O_h_ of puma (R^2^ = 0.00, p = 0.818, n = 30). Relative humidity, however, did not affect δD_h_ of bobcats (R^2^ = 0.05, p = 0.146, n = 44) and puma (R^2^ = 0.068, p = 0.164, n = 30) (Table S2). The isotope composition of the analyzed hair samples spanned a range of 99.3 ‰ for δD_h_ and 12.6 ‰ for δ^18^O_h_ in bobcat, and 95.4 ‰ for δD_h_, and 18.2 ‰ for δ^18^O_h_ in puma (Figures 2 and 3). No significant relationship was found between δD_h_ and δD_riv_ for both species (bobcat: R^2^ = 0.005, p = 0.65, n = 44; puma: R^2^ = 0.040, p = 0.291, n = 30) (Figure 2). Likewise δ^18^O_h_ and δ^18^O_riv_ were not significantly correlated (bobcat: R^2^ = 0.030, p = 0.261, n = 44; puma: R^2^ =  0.055, p = 0.211, n = 30) (Figure 3). No effect of sex on the isotopic relationship between hair and water was observed for both species (Table S2). There was a weak correlation between δD_h_ and δ^18^O_h_ values of the same hair samples in bobcat (R^2^ = 0.195, p = 0.003, n = 43) but not in puma (R^2^ = 0.0002, p = 0.939, n = 30) (Figure 4). Results for the hair isotope compositions of cottontail rabbits exhibited a strong δD_h_–δD_riv_ (δD_h_: R^2^ = 0.81, p<0.0001, n = 13) and a moderate δ^18^O_h_–δ^18^O_riv_ (δ^18^O_h_: R^2^ = 0.25, p = 0.083, n = 13) positive relationship (Figures 2 and 3). The eastern cottontail rabbits also displayed a significant positive correlation between δD_h_ and δ^18^O_h_ values of the same hair samples (R^2^ = 0.571, p = 0.003, n = 13) (Figure 4).

## Discussion

Both puma and bobcat lacked the expected correlation between water isotopes in local water and hair, and also exhibited a complete decoupling between δ^18^O_h_ and δD_h_. This finding contrasted strongly with results from numerous previously published studies on keratin tissues of omnivores and herbivores. Hence, tracing the provenance of feline carnivores such as puma and bobcat based on δ^18^O_h_ and δD_h_ isoscapes does not appear to be possible, as individuals could not be reliably placed on δ^18^O_p_ and δD_p_ maps. Potential explanations for this lack of correlation between hair and ambient water isotope compositions are discussed below.

### Can relative humidity affect carnivore δ^18^O_h_ and δD_h_?

In our study, relative humidity showed a significant modest effect on δ^18^O_h_ of bobcats (R^2^ = 0.21, p = 0.002) but not on puma (R^2^ = 0.00, p = 0.818) (Table S2). Previous studies on mammalian bone phosphate showed that relative humidity controls the δ^18^O_bp_ values of herbivore species with low drinking water requirements (e.g. [30]). For example, δ^18^O_bp_ values of Australian macropods [37], rabbits and hares [44] have been shown to correlate strongly with changes in relative humidity independent of δ^18^O_p_, whereas the δ^18^O_bp_ of North American deer [38] were influenced by both relative humidity and δ^18^O_p_. Low humidity increases the rate of evaporation of surface water and evapotranspiration of leaf- and grass-water and thus leads to oxygen isotopic enrichment effects in plants [66], [67]. Drought-tolerant animals who obtain most of their water from plants thus reflect levels of environmental humidity, in particular their δ^18^O_bp_ increases with decreasing relative humidity. However, Kohn [30] hypothesized that the importance of relative humidity diminishes with increasing trophic level. Our data support Kohn's hypothesis that predators are less controlled by relative humidity than herbivores. Bobcat δ^18^O_h_ compositions were weakly affected by relative humidity (R^2^ = 0.21, p = 0.002), most likely because they prey upon rabbits, whose δ^18^O_bp_ compositions are humidity dependent (R^2^ = 0.86; [44]). In contrast, puma δ^18^O_h_ compositions were not influenced by relative humidity (R^2^ = 0.00, p = 0.818), probably because they feed on white-tailed deer, whose δ^18^O_bp_ is affected by both relative humidity and δ^18^O_p_ [38]. Unlike oxygen isotopes, δD_h_ values of both feline carnivores were not influenced by relative humidity (bobcat: R^2^ = 0.05, p = 0.15; puma: R^2^ = 0.07, p = 0.16). Similar observations were made for δD_bc_ (bone collagen) of white-tailed deer by Cormie et al. [68]. We conclude that relative humidity particularly affects δ^18^O_t_ of predators (e.g. bobcats) that feed on drought -tolerant herbivore species like rabbits. However, relative humidity did not explain the lack of a correlation between δD_h_-δ^18^O_h_ observed in both felids we studied.

### Does an isotopic disequilibrium between food and water affect δD_h_?

It was documented previously [13], [27], that δD_h_ is not well correlated with δD_p_, if (i) ingested food or water sources (e.g. exotic foods, marine-based diet, high altitude food or snow melt drinking water) are not isotopically related to local meteoric water and/or (ii) migration between isotopically distinct habitats takes place. We tested whether the ingested food sources (i.e. key prey species) of bobcat and puma were in disequilibrium with δD_p_, and so caused the lack of a correlation between H/O isotopes in precipitation and those in felid hair. In North America, the preferred prey species of puma is the white-tailed deer (*Odocoileus virginianus*) [69], whose δ^18^O of bone phosphate (δ^18^O_bp_) [38] and δD bone collagen values (δD_bc_) [56] strongly correlate with δ^18^O_p_ and δD_p_, respectively (Figure 2 and 3). In contrast, bobcats mainly prey on lagomorphs [70], whose δ^18^O_h_ and δD_h_ values we also found to show a direct relationship with δ^18^O_p_ and δD_p_ (Figure 2 and 3). Thus the oxygen and hydrogen isotopic composition of prey are not reflected in the hair of their respective predators. Cats are not obligate drinkers [71] and hence isotopic content of drinking water does not explain the lack of a correlation between δD_p_ and δD_h_ in felines.

Migration between isotopically distinct biomes during biosynthesis of hair might also affect the correlation of δD_h_ with δD_p_. We would have expected this effect based on potential species- or sex-specific behavioral differences characterizing our study species. Puma and bobcat, for instance, have significantly different home range sizes [2], [72], which are also known to vary between seasons and sex. Although carnivores exhibit typical mammalian dispersal behaviour, where males disperse and females are philopatric [73]; we did however not observe an effect of sex on the hair/water isotope correlation for both carnivore species (Table S2). We therefore concluded that the isotopic disequilibrium of food and water does not explain the lack of a relationship between δD_h_ and δD_p_ observed in puma and bobcats.

### Does a carnivorous diet affect δD_h_?

Some studies have suggested a dietary trophic-level effect on H isotope systematics of animal tissues [13], [42], [45], [74], [75]. Possibly, high levels of animal protein consumption leads to a decoupling of δD in keratins from δD_p_ and a deviation from the mean relationship between keratin δD and δ^18^O [45], [76]. Diet may thus represent a confounding factor in the use of H and O isotopes for geographic tracking [13].

**Figure 5:**
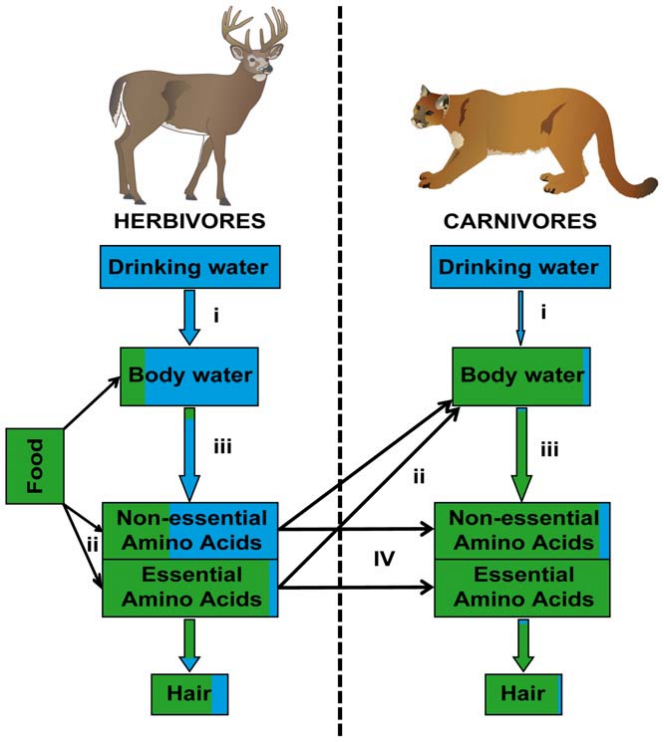
Hydrogen isotope model of herbivores and carnivores. Model of hydrogen isotope physiology and the contribution of food and water to non-exchangeable hydrogen in the hair of herbivores and carnivores. Letters represent processes where isotope fractionation occurs (see text for detailed discussion). Blue coloring represents water inputs and green food inputs.

We developed a simple model of hydrogen isotope incorporation in carnivores to illustrate possible trophic-level enrichment and isotopic decoupling of δD_h_ in carnivores. Various fractionation factors and source pools contributing to non-exchangeable hydrogen in hair were considered (Figure 5). Controlled experiments on domestic cats have shown that, on average, only 1% of their total water input originates from drinking water [71]. So, drinking water likely has minor control on deuterium enrichment in felids, leaving the isotopic input of prey as a major determinant of the isotopic signature of carnivore body water. In this aspect, strict carnivores differ significantly from herbivores and omnivores, whose body water is to a large extent (64–80%, see Table 1) obtained from drinking water (Figure 5(i)). Isotope fractionation from drinking water to body water occurs [35], [42], [77] and may play an important role in δD_h_ enrichment of carnivore proteins. Feline carnivores consume prey species whose δD_bw_ and δ^18^O_bw_ are expected to be higher than δD_p_ and δ^18^O_p_ due to evaporative enrichment from insensible water loss through skin and breath vapor loss [34], [78]. Consequently, carnivores mainly consuming deuterium-enriched prey should have higher δD_bw_ values over those of their prey. A similar process has been documented in humans for the consumption of cow milk and the resulting enrichment in deuterium of consumer tissue [42], [79]. Otherwise the consumption of D-depleted prey might decrease the carnivore δD_bw_ values particularly during winter when prey species have built up their body fat reserves. Fat reserves are known to have significantly more negative δD values than proteinaceous tissues [24], [76], [80], [81]. The temporary alternation of D-depleted and -enriched carnivore diets relative to δD_p_, based on differential seasonal consumption of lipids and proteins, respectively, might change the δD_bw_ [35] and is finally recorded in δD_h_ during carnivore hair growth [82].

**Table 1:** Food and drinking water inputs of hydrogen in the body water of different organisms under laboratory conditions.

Species	Food (%)	Drinking water (%)	Reference
Lab rats	37	64	[118]
Woodrats	29	71	[17]
Doves	15	85	[33]
Humans	20	80	[34]
European roe deer	24	76	[119]

Hydrogen isotope fractionation can also occur during the oxidation of food to form body water (see Figure 5 (ii)). Carnivores have the ability to digest and utilize high levels of dietary fat and protein and so produce relatively higher levels of metabolic water [54], [83], [84]. Catabolism of macronutrients and production of metabolic water could cause hydrogen isotope fractionation processes leading to deuterium enrichment [35], [41]. In addition, isotopic fractionation most likely happens during the incorporation of body water into tissue amino acids (see Figure 5 (iii)). Water from food, drinking water and metabolism are the three source pools which can be fixed into newly synthesized non-essential amino acids [13]. However, the fraction of hydrogen fixed into amino acids may scale with the extent of non-essential amino acid synthesis in the body. This, in turn, is related to the level and amino acid composition of dietary protein intake [85]. Carnivores exhibit low levels of non-essential amino acid synthesis because their natural meat-rich diet contains all required amino acids [86]. Consequently, low levels of hydrogen fixed into amino acids in vivo could maximize the transfer of hydrogen from diet to hair thereby enhancing the contribution of isotopically heavy, prey-derived hydrogen in carnivore hair [13]. Finally, it is also possible that isotope fractionation occurs during the transfer of food amino acids to tissue amino acids (Figure 5 (iv)). δD_h_ enrichment of carnivore proteins could also occur through selective catabolism of isotopically lighter amino acids [45]. We conclude that there are several possible isotopic fractionation steps during the metabolic incorporation of hydrogen into carnivore hair that could induce enrichment in deuterium and leading to higher δD_h_ and a loss of correlation with δD_p_.

### Effects of carnivore physiology and metabolism on δD_h_ and δ^18^O_h_

If diet rather than drinking water solely controls carnivore δD, we would have expected a variation of the hair/water regression in slope and intercept compared to herbivores and omnivores. Because there was no significant correlation between oxygen and hydrogen isotope compositions of hair and precipitation and δD_h_ and δ^18^O_h_, we therefore suspected the dietary trophic-level effect was potentially obscured by physiological and metabolic adaptations in carnivores [87]. Animals which display deviations from the normal covariance between δD and δ^18^O values in keratin are carnivorous fish, birds and mammals [45] and ancient human populations with a meat-rich diet [13], [42], [74], which all consume high levels of animal protein and fat. From a purely nutritional perspective, they are all strict carnivores. Through evolution, their adherence to a specialized meat-rich diet induced changes in their metabolic pathways and nutritional requirements [54]. These physiological and metabolic adaptations in strict carnivores could considerably affect the H and O isotope systematics of their keratins.

The H and O isotope compositions of human hair strongly covary, and are closely related to meteoric (drinking) water at the place of residence [8] with the exception of mid 20^th^ century Inuit people [13]. Bowen et al. [13] did not find strong support for ubiquitous effects on the H/O isotope systematics of human hair related to physiological adaptations. However, in pre-globalization times, the typical diet of the Inuit contained high levels of dietary protein and fat from high trophic-level marine animals [88]. Mid 20^th^ century Inuit people thus fed at the highest trophic level of all humans. Since marine food webs have typically longer chain lengths than terrestrial food webs [89], the consumption of marine predators may confer a trophic-level enrichment of Inuit δD_h_ [13]. Historic Inuit are also classified as obligate carnivores among omnivorous humans because they require nutrients that are present only in animal tissue of their diet [90] and so differ from other ancient humans who used a marine-dominated but omnivorous diet like the Ainu from Japan and Thai from Thailand [13].

Measurements of δD in feathers have been successfully applied in many bird species to estimate the origins of migrating and wintering individuals [36]. However, in strictly carnivorous raptors like Amur Falcons (*Falco amurensis*; [91]) and Cooper's Hawks (*Accipiter cooperii*; [92]) the linkage between feather δD and δD_p_ was weaker [9], [93]. However, this may be complicated due to the fact that several raptors grow feathers during periods of high work associated with breeding and so may produce more deuterium enriched feathers due to evaporative water loss.

The natural diet of wild felids contains a high proportion of the energy as protein, a variable percentage as fat and a very low percentage as carbohydrate [55]. Metabolic adaptations mainly concern the loss of anabolic pathways required for the synthesis of nutrients universally present in their natural meat-based diet [94]. One of the most striking aspects here is that strict carnivores have lost the ability to produce metabolic compounds that are commonly synthesized by virtually all herbivores and omnivores. For example, cats lack the enzymatic machinery to synthesize some amino and fatty acids, thereby significantly increasing their basal requirement for proteins and essential amino acids. When ingesting prey, wild cats avoid consuming plant materials contained in the intestines [87] and hence the digestion of dietary starches and sugars has adapted to low carbohydrate intake [95].

Currently we lack a testable explanation for our observed and confounding isotopic patterns, but considering the unique felid physiology, we hypothesized that the food metabolism of strict carnivores may exert a vital effect particularly on δD_h_. This may also affect the relative contributions of all sources to protein synthesis and hair formation. Recent findings from Pecquerie et al. [41] support our hypothesis. They propose two mechanisms involved in stable isotope fractionation during metabolic reactions: First, the selection of molecules for the anabolic or the catabolic pathway routes depends on their isotopic composition. Second, the concept of atom recombination recognizes that molecules are not completely disassembled into elements during chemical reactions [96]. A non-random allocation of atoms of a particular substrate (e.g. food amino acids) to a particular product (e.g. keratin amino acids) impacts isotopic composition of a given product (e.g. hair). While isotope fractionation takes place in metabolic reactions [41], these were particularly modified during the evolutionary history of carnivores. Knowing that approximately two thirds of the hydrogen in human hair are derived from food [27], we suspect that carnivores might be affected by alternate modes of isotopic routing of macronutrients into hair (Table 2).

**Table 2:** Food and drinking water inputs of hydrogen in hair and feathers of different organisms.

Species	Food (%)	Drinking water (%)	Reference
Woodrats	75	25	[17]
Japanese quail	74–69	26–32	[24]
House sparrow	82	18	[21]
Humans	69, 64^a^, 73^b^	31, 36^a^, 27^b^	[27]

a Data after [15]

b Data after [8]

The water metabolism in feline carnivores also differs from herbivores and omnivores. Cats drink to a limited extent [55], [83] and excrete concentrated urine [97]–[99]. In addition they produce relatively high levels of metabolic water, which contributes on average 10% to their total water intake [54], [83]. Drinking water volume, however, exerts a significant physiological control on the isotopic composition of hydrogen and oxygen in human body water [26] (Table 1). Besides various water conservation adaptations, strict carnivores have higher basal metabolic rates than other mammals [100], [101]. A high metabolic rate associated with a low rate of drinking, results in a weak correlation of δ^18^O_bp_ with δ^18^O_p_ [25]. We infer that this applies to strict carnivores and assumed that relatively smaller contributions of oxygen in carnivore hair originate from drinking water. In addition, cats lose water primarily through panting [102] vs. from sweat glands of foot pads [103]. Differences in the isotope compositions of liquid water during sweating vs. vapor during panting should affect their body isotopic compositions. Panting animals should thus have higher δ^18^O_bw_ and δ^18^O_h_ values than animals that sweat because water vapour lost in panting is more depleted in ^18^O [30], [104]. The same should apply to δD_bw_ and δD_h_.

In contrast to the weak correlation between feline carnivore hairs δD_h_ and δ^18^O_h_ and meteoric water δ^18^O_p_ and δD_p_ (Figures 2 and 3), a good correlation between claw δD_c_ and δD_p_ was observed in a recently published study of migrating pumas in the USA [6]. The reason why the two keratineous tissues do not reflect meteoric water values in the same way remains unclear. However, a similar paradox is known for human fingernails and hair, with nails displaying a more variable H/O isotope composition and a comparatively weaker correlation between δD_c_ and δD_water_ (R^2^ = 0.6) compared to hair (R^2^ = 0.9) from the same individuals [14], [53]. The reverse trend in feline carnivores may result from different formation rates of hairs [63] and nails [105], alternate modes of isotopic routing of macronutrients into hair and nail as well as different amino acid compositions of hair and nail [106].

### Amino acid composition of cat hair

The isotopic values of keratins are generally defined by the isotopic composition of their constituent amino acids [106]. For example, cysteine, serine and glutamate, all non-essential, metabolically active amino acids are present at very high proportions in hair [107]. Their isotopic composition reflects both food and drinking water, with a slight bias towards food. Due to the high relative abundance of non-essential amino acids, their isotope composition can often dominate the bulk H and O isotope hair signature and mask the isotope composition from essential amino acids. The latter are present at lower proportions and routed directly from dietary sources [108]. The constancy of amino acid composition and hence isotopic values between tissues, even for related proteins like nail and hair, cannot be implied [106]. Large isotopic differences between amino acids of different components have been observed [109]–[111], reflecting their formation via different metabolic, synthetic and catabolic processes. However, the amino acid composition of cat hair protein is comparable with that of dog, horse, sheep and human hair [107]. Apparently only the proline content of cat hair protein appears to be lower and glycine appears to be higher than in the other species [107]. Variations in amino acid composition of cat hair might thus be responsible for some of the differences in isotopic patterns we have observed.

### Does tanning of museum skins have an effect on the H/O isotopic composition of hairs?

To our knowledge this is the first H/O isotope study on mammal hair which benefits from large museum collections as a valuable source of sample material. However, it has not been assessed whether the tanning process used for preserving hides affects the H/O isotopic composition of taxidermy skins. Tanning chemicals are intended to stop deterioration processes of the skin. At a molecular level tanning chemicals act as solid spacers, which replace the H bonds linking the polypeptide chains of the collagen fiber and thus stabilize the collagen structure of museum skins [112]. Collagen and hair are both proteinaceous tissues and interpeptide H-bonding is abundant and important for maintaining the alpha-helical structure of collagen and hair [113]. Thus, tanning chemicals could potentially alter the non-exchangeable H isotope composition of hairs. However, we hypothesize that tanning chemicals did not affect the H/O isotopic composition of the analyzed felid hairs. First, the rabbit hairs which have most likely undergone the same tanning process as felid hides, showed good isotopic (δD_h_ and δ^18^O_h_) correlation between hair and meteoric waters (Figure 2 and 3). Second, initial results from a small “before and after tanning experiment” using a common mineral tanning technique (aluminium salts [114]) on hairs from different mammal species indicated that there was no significant effect of the tanning process on the H isotopic values of these hair samples (data not shown).

### Conclusions

Stable isotope (H, O) data from bobcat and puma hairs from a range of locations across North America revealed that feline carnivores cannot be placed on δ^18^O and δD isoscapes for forensic investigation purposes. The effective application of water isoscapes for geographic source determination of feline carnivores is most likely compromised by major controls of their diet, physiology and metabolism on δ^18^O_h_ and δD_h_. However, we noted that the integration of H and O isotopes into animal proteins in general remains poorly understood. Isotope fractionation and routing during metabolic and tissue formation processes is complex and presumably varies between herbivores, omnivores and carnivores. Significant research thus remains to be performed to characterize the precise origin and sensitivities of the observed isotope signals. Controlled feeding experiments on strict carnivores like domestic cats are now needed to track isotope routing of macronutrients and their incorporation into different tissue types (e.g. [17], [24]). With the objective to enhance the resolution of H and O isotope analysis of proteins, we suggest compound-specific single amino acid isotope analysis may give improved insights into isotope fractionation processes during protein, and by a comparative isotope analysis of essential versus non-essential amino acids. To date most studies have used bulk tissue protein isotopic values of hydrogen and oxygen [8], [13], [20] but little research has been conducted at the level of single amino acids in hair that was limited to C, N and S isotopes [115]–[117]. Unfortunately, there are no reported applications of hair δ^18^O and δD compound-specific isotope analysis of amino acids. This represents an important area of future research and will contribute to a better understanding of the observed variations in bulk protein H and O isotope ratios.

## Supporting Information

Table S1 **Sample list.**
(XLS)

Table S2 **Statistical analysis.**
(DOC)
