# Supplementary material for: Tracking Cats: Problems with Placing Feline Carnivores on δ18O, δD Isoscapes
Source: PLoS One. 2011 Sep 9;6(9):e24601. doi: 10.1371/journal.pone.0024601 (PMC3170367; doi:10.1371/journal.pone.0024601)
Supplement: Table S2 — Statistical analysis. (DOC) [file pone.0024601.s002.doc]

| **Table S2. Statistical analysis** |  |  |  |  |  |  |  |  |
| --- | --- | --- | --- | --- | --- | --- | --- | --- |
| **Regression equations** |  | **slope** | **SD (1s)** | **intercept** | **SD (1s)** | **r²** | **p-value** | **n** |
| **δ^18^O_hair_ = f(δ^18^O_river_)** | All cats | 0.010 | 0.095 | 12.920 | 1.136 | 0.0002 | 0.914 | 74 |
|  | All bobcats | 0.104 | 0.091 | 14.964 | 1.056 | 0.030 | 0.261 | 44 |
|  | All pumas | -0.232 | 0.181 | 8.543 | 2.267 | 0.055 | 0.211 | 30 |
|  | All rabbits | 0.298 | 0.156 | 18.394 | 1.228 | 0.248 | 0.083 | 13 |
|  | All deer | 0.588 | 0.072 | 22.096 | 0.806 | 0.699 | < 0.0001 | 31 |
|  | Female bobcats | 0.007 | 0.121 | 14.018 | 1.349 | 0.0002 | 0.953 | 22 |
|  | Male bobcats | 0.152 | 0.162 | 14.974 | 2.120 | 0.059 | 0.363 | 16 |
|  | Female pumas | -0.186 | 0.204 | 9.190 | 2.321 | 0.085 | 0.385 | 11 |
|  | Male pumas | 0.615 | 0.733 | 20.517 | 10.266 | 0.055 | 0.418 | 14 |
|  | Female rabbits | 0.535 | 0.188 | 19.118 | 1.538 | 0.618 | 0.036 | 7 |
|  | Male rabbits | 0.009 | 0.030 | 18.606 | 0.219 | 0.046 | 0.785 | 4 |
| **δD_hair_ = f(δD_river_)** | All cats | 0.052 | 0.073 | -68.699 | 6.633 | 0.007 | 0.479 | 74 |
|  | All bobcats | 0.042 | 0.091 | -74.343 | 8.015 | 0.005 | 0.650 | 44 |
|  | All pumas | 0.129 | 0.119 | -54.855 | 11.332 | 0.040 | 0.291 | 30 |
|  | All rabbits | 0.797 | 0.116 | -25.457 | 6.599 | 0.810 | < 0.0001 | 13 |
|  | All deer | 0.856 | 0.063 | 20.284 | 4.383 | 0.866 | < 0.0001 | 31 |
|  | Female bobcats | 0.074 | 0.120 | -74.082 | 10.342 | 0.019 | 0.542 | 22 |
|  | Male bobcats | 0.114 | 0.183 | -62.123 | 17.944 | 0.027 | 0.544 | 16 |
|  | Female pumas | 0.138 | 0.199 | -47.662 | 17.226 | 0.051 | 0.505 | 11 |
|  | Male pumas | -0.181 | 0.422 | -91.547 | 44.707 | 0.015 | 0.675 | 14 |
|  | Female rabbits | 0.909 | 0.176 | -23.537 | 10.346 | 0.843 | 0.004 | 7 |
|  | Male rabbits | 0.622 | 0.164 | -24.871 | 8.745 | 0.878 | 0.063 | 4 |
| **δD_hair_ = f(δ^18^O_hair_)** | All cats | -1.641 | 0.691 | -52.438 | 9.285 | 0.074 | 0.020 | 73 |
|  | All bobcats | -3.457 | 1.095 | -30.451 | 15.607 | 0.195 | 0.003 | 43 |
|  | All pumas | 0.072 | 0.936 | -66.977 | 11.370 | 0.0002 | 0.939 | 30 |
|  | All rabbits | 8.734 | 2.282 | -204.308 | 38.210 | 0.571 | 0.003 | 13 |
|  | All deer | 7.850 | 0.636 | -159.827 | 10.459 | 0.840 | < 0.0001 | 31 |
|  | Female bobcats | -3.221 | 1.574 | -36.655 | 22.494 | 0.181 | 0.055 | 21 |
|  | Male bobcats | -4.003 | 2.218 | -19.822 | 29.831 | 0.189 | 0.093 | 16 |
|  | Female pumas | 2.238 | 2.309 | -82.727 | 26.800 | 0.095 | 0.358 | 11 |
|  | Male pumas | -0.886 | 0.993 | -61.864 | 12.925 | 0.062 | 0.390 | 14 |
|  | Female rabbits | 10.889 | 1.964 | -233.724 | 31.183 | 0.860 | 0.003 | 7 |
|  | Male rabbits | 54.724 | 71.022 | -1064.063 | 1318.024 | 0.229 | 0.522 | 4 |
| **δ^18^O_hair_ = f(δ^18^O_precip_)** | All cats | 0.020 | 0.129 | 13.022 | 1.494 | 0.000 | 0.880 | 74 |
| **δD_hair_ = f(δD_precip_)** | All cats | 0.030 | 0.103 | -70.553 | 8.761 | 0.001 | 0.773 | 74 |
| **δD_hair_ = f(δD_mean summer precip_)** | All pumas | 0.216 | 0.231 | -51.359 | 16.394 | 0.030 | 0.358 | 30 |
| **δ^18^O_hair_ = f(δ^18^O_mean summer precip_)** | All pumas | -0.597 | 0.362 | 6.024 | 3.269 | 0.088 | 0.110 | 30 |
| **δD_hair_ = f(δD_mean spring precip_)** | All pumas | 0.143 | 0.147 | -53.948 | 13.297 | 0.03 | 0.34 | 30 |
| **δ^18^O_hair_ = f(δ^18^O_mean spring precip_)** | All pumas | -0.385 | 0.216 | 6.773 | 2.642 | 0.102 | 0.086 | 30 |
| **δD_hair_ = f(δD_annual_ _precip_) with rel. humidity (h)** | All pumas |  |  |  |  | 0.068 | 0.387 | 30 |
| **δ^18^O_hair_ = f(δ^18^O_annual_ _precip_) with rel. humidity (h)** | All pumas |  |  |  |  | 0.115 | 0.193 | 30 |
| **δD_hair_ = f(δD_river water_) with rel. humidity (h)** | All pumas |  |  |  |  | 0.075 | 0.350 | 30 |
| **δ^18^O_hair_ = f(δ^18^O_river water_) with rel. humidity (h)** | All pumas |  |  |  |  | 0.060 | 0.436 | 30 |
| **δD_hair_ = f(rel. humidity (h))** | All pumas | 0.634 | 0.444 | -100.719 | 24.549 | 0.068 | 0.164 | 30 |
| **δ^18^O_hair_ = f(rel. humidity (h))** | All pumas | -0.022 | 0.093 | 12.410 | 5.131 | 0.002 | 0.818 | 30 |
| **δD_hair_ = f(δD_mean summer precip_)** | All bobcats | 0.038 | 0.157 | -75.381 | 9.918 | 0.001 | 0.812 | 44 |
| **δ^18^O_hair_ = f(δ^18^O_mean summer precip_)** | All bobcats | 0.269 | 0.157 | 15.969 | 1.297 | 0.066 | 0.093 | 44 |
| **δD_hair_ = f(δD_mean spring precip_)** | All bobcats | 0.035 | 0.110 | -74.901 | 9.204 | 0.002 | 0.751 | 44 |
| **δ^18^O_hair_ = f(δ^18^O_mean spring precip_)** | All bobcats | 0.141 | 0.103 | 15.358 | 1.168 | 0.043 | 0.176 | 44 |
| **δD_hair_ = f(δD_annual_ _precip_) with rel. humidity (h)** | All bobcats |  |  |  |  | 0.09 | 0.143 | 44 |
| **δ^18^O_hair_ = f(δ^18^O_annual_ _precip_) with rel. humidity (h)** | All bobcats |  |  |  |  | 0.207 | 0.009 | 44 |
| **δD_hair_ = f(δD_river water_) with rel. humidity (h)** | All bobcats |  |  |  |  | 0.104 | 0.105 | 44 |
| **δ^18^O_hair_ = f(δ^18^O_river water_) with rel. humidity (h)** | All bobcats |  |  |  |  | 0.213 | 0.007 | 44 |
| **δD_hair_ = f(rel. humidity (h))** | All bobcats | -0.502 | 0.339 | -47.898 | 20.370 | 0.05 | 0.146 | 44 |
| **δ^18^O_hair_ = f(rel. humidity (h))** | All bobcats | 0.127 | 0.038 | 6.391 | 2.305 | 0.206 | 0.002 | 44 |
| **δD_river_ = f(δ^18^O_river_)** |  | 7.883 | 0.130 | 4.370 | 1.561 | 0.981 | <0.0001 | 75 |

**P-value: Probability value; p-values < 0.05 were considered statistically significant.**

**SD: Standard deviation**

**N: Number of observations**

**SD: Standard deviation**

**R²: Coefficient of determination**

**δ^18^O_hair_: Oxygen isotope composition of hair**

**δD_hair_: Hydrogen isotope composition of hair**

**δD_precip/river_: Hydrogen isotope composition of precipitation/ river water**

**δ^18^O_precip/river_: Oxygen isotope composition of precipitation/ river water**

**h: relative humidity (%)**
